# Notes and Comments

**Published:** 1922-10

**Authors:** 


THE
### HOSPITAL and HEALTH
REVIEW

### THE LOCHMAREE OUTBREAK OF FOOD POISONING.

AT Lochmaree, in the Highlands, eight persons died because, in one, or possibly two, small jars of wild-duck paste a microscopic germ had grown and produced a deadly poison—a toxin, as it is termed. In Britain this is the first recorded outbreak of this type of food poisoning. The germ, which is called the *bacillus botulinus*, was discovered by a Belgian, Van Ermengem, in 1896. Twenty-three persons were taken ill after eating ham that had been preserved in brine, and three of them died. From the tainted food Van Ermengem was able to isolate the *bacillus botulinus*, and, by using cultures of the germ, he was able to produce the same symptoms in animals. There have been many outbreaks on the Continent of Europe, and several in America; but its relative rarity is shown by the American figures produced by Rosenau in a recent article. "The high mortality, the distressing symptoms, and the relation to food have dramatic news value, and the disease has recently caused concern and alarm out of all proportion to its prevalence. A disease which, during a period of 22 years, has made only about 150 people ill, and caused the death of 111, among approximately a hundred millions of people, cannot be compared in magnitude to tuberculosis, syphilis, and other public health problems." Formerly, sausages were supposed to be the main source of botulism. It is true that the foods concerned in outbreaks have been for the most part meats, such as sausage and ham, but in America, other foods have been involved, such as string beans, cottage cheese, corn, asparagus, salad, spinach, and ripe olives. Botulism has also been attributed to turkey, beef, chicken and fish.

The *bacillus botulinus* produces its toxin by growing on food, whether animal or vegetable. As it does not grow where oxygen is present, a sealed tin of preserved food, if accidentally or otherwise inoculated with the bacillus, is an ideal culture medium. The germ goes on generating its poison out of the materials of food, and may then itself die; but the poison remains, and, without any obvious sign of its presence, may, as in this outbreak, produce its specific effects. Thus in food poisoning by *bacillus botulinus* the person is killed not directly by the bacillus itself, but by the poison it has previously produced in the food. Botulism, therefore, would be classified not as an infection, but as an intoxication.

The victim begins to show symptoms from 16 to 36 hours after partaking of the poisonous food. In some of the cases of the recent outbreak the symptoms appeared earlier, in some later; but in none later than 40 hours after ingestion. Among the earliest symptoms are weakness, dizziness, headache, and perhaps nausea and sickness. There is difficulty of vision, the patient sees double. The eyelids droop, the pupils are dilated, and at a certain stage do not react to light. These conditions are all due to the effect of the toxin on the nerves. The bowel gets more or less paralysed, and there is constipation. The lips become paralysed, and there is difficulty of speech. The muscles about the throat are paralysed, and there is difficulty of swallowing. The voice muscles become paralysed, and there is loss of voice. The tongue is paralysed and cannot be protruded. The respiration becomes paralysed, and the patient gasps for breath. Ultimately the paralysis of the respiration and of the heart becomes complete, and the patient dies. Meanwhile, the temperature remains normal or sub-normal, unless there are special complications. In this respect, poisoning by this toxin differs from the ordinary infectious diseases, where there is always a rise of temperature at some stage. There is no pain, although the distress due to the various paralyses may be great; and the mind remains clear to the last.

At the official inquiry into the deaths of the eight persons it was shown that the wild-duck paste had been made in London by a firm of the highest standing. Dr. MacFadden, Senior Medical Officer of the Food Section of the Ministry of Health, inspected the factory and found everything satisfactory. But he considered that bacteriological sterilisation was very difficult to achieve. The manager of the factory stated that about 700 jars of paste were made at the same time as the poisonous one and disposed of, and probably had all been used. All the firm's products were packed in airtight vessels of tin or glass and finally heated to 210° to 212°F. for 45 minutes and, in the case of game, to 214° to 216° for 50 minutes. Though one million jars of meat paste were sold yearly there never had been any complaint before.

The jury found that no fault or negligence was attributable to any one and that it was not possible to take any precautions by which the accident might have been avoided. They thought it desirable that every vessel containing preserved meat, fish, fruit, or vegetables should bear a distinctive mark by which the details of manufacture may be traced.

In order that materials from such outbreaks shall be fully subjected to laboratory methods, the Medical Research Council, which is at the service of all the departments, has arranged that its specialists shall investigate all material of this order submitted to them. The Council established in 1921 a Canned Foods Committee, on which Dr. MacFadden represents the Ministry of Health, and Dr. Gerald Leighton the Scottish Board of Health. Through this Committee the Council can proceed instantly with the investigation of any outbreak in the United Kingdom. Antitoxin both for diagnostic and therapeutic purposes has been prepared, but, as in the case of lockjaw (tetanus), it is of little value after the symptoms are well established. To be of service it must be given early. The Ministry of Health is depositing supplies of botulism anti-toxin at sixteen centres, chosen so that the remedy shall be readily available (say, within two hours by motor of any district). These centres are Portsmouth, Plymouth, Bristol, Cardiff, Aberystwyth, Birmingham, Manchester, Liverpool, Leeds, Carlisle, Newcastle, Nottingham, Norwich, Cambridge, Oxford and Hull. To this list will be added any other district on application from its medical officer of health. Arrangements are in hand for securing the storage of the antitoxin under suitable conditions as regards temperature and light, and for periodical withdrawal and renewal to prevent deterioration.

### UNHYGIENIC FOOD FACTORIES.

It is hoped that the lamentable tragedy of Loch-maree will have the result of providing adequate regulations for the inspection of places where food for human consumption is prepared or kept for sale. Although in this case it would appear from the evidence given at the inquiry that the food pastes in question were prepared under model conditions, there are many cases in the country where the conditions are unsatisfactory in the extreme. Generally speaking, in the larger factories the materials used are sound and wholesome, the premises are clean and great care is taken to avoid contamination. But there are old sheds and stables, and even cellars, which are considered good enough at the present time for making sausages, meat pies, potted meats and other foods. According to an official report by Dr. A. W. J. MacFadden, who gave evidence at Dingwall as the senior medical officer of the Food Section of the Ministry of Health, "ordinary practices of cleanliness in such places are generally entirely absent. The machines and utensils used were, as a rule, dirty and uncared for and the persons and clothing of those employed were in a similar condition. . . . Evidence of the access of rats and mice to stored food materials and to utensils and tables used in the preparation of food was frequently observed by the discovery of rodents' droppings upon them or in their vicinity, and in some cases the firms did not even trouble to remove the evidence in question before resuming work."

This report contained detailed evidence acquired in the supervision of factories during the war, in the interests of the soldier; but at the present time the powers which local authorities possess under the Public Health Acts are inadequate for securing a proper standard of suitability and cleanliness of premises. By means of systematic inspection during the war, hygienic conditions were kept on a fairly satisfactory level, but it is feared by those who know that in many cases some of the smaller and less reputable firms have allowed their premises to drift into the old condition. As our industrial population becomes more and more dependent for nourishment on prepared and cooked foods, it is increasingly urgent that there should be more stringent regulations to improve this class of premises.

### MILK ORDERS.

The new regulations for carrying into effect the Milk and Dairies Amendment Act of 1922 will be of special interest to sanitary inspectors and others. Section 1 provides for the postponement of the 1915 Act until September 1, 1925. Section 2 empowers the local authority under certain conditions to refuse to register a retail purveyor of milk or to remove such person from the register. This necessitates two registers being kept, one restricted to retail purveyors and the other containing all other persons, wholesale traders and producers, who do not sell milk by retail. The course to be taken will generally be initiated by a medical officer or sanitary inspector presenting a report to draw attention to any act or default on the part of the retailer, and showing in what respect the public health is or is likely to be endangered thereby.

Section 3 continues with modifications the provisions for the present grading of milk. The existing orders are continued in force until January 1 next. After that there will be a new system of grading. Fees are to be paid for licences, so as to cover the cost of necessary inspection and bacteriological examinations. Conditions as to certified milk will be substantially the same as those at present applying to Grade A (certified) milk, and the licence for this grade will continue to be issued directly by the Ministry of Health. Local authorities will be empowered to issue licences for Grade A milk and for "Pasteurised" milk, but the general conditions for this have not yet been laid down.

Section 4 enables local authorities to deal with cases in which additions are made for fraudulent purposes to milk intended for sale. Section 5 imposes a heavy penalty on any person who sells the milk of a cow suffering from tuberculosis of the udder, where it is proved that he knew or could have ascertained by ordinary care that the cow was suffering from that disease. Sections 6 and 7 lay down procedure, but Section 8 is important to all concerned in the inspection of foods. It requires the Minister of Health to make regulations as regards imported milk, and also empowers him to prescribe standards for dried and condensed milks and the manner in which receptacles containing dried, condensed, skimmed or separated milk are to be labelled or marked. The remaining sections deal with penalties and proceedings and other legal points.

Sir Alfred Mond has called the attention of sanitary authorities to the very wide powers which they already possess for the sanitary control of dairies and cowsheds, and has stated that in his opinion, "by the careful and systematic exercise of existing powers, together with the additional powers afforded under the new Act, local authorities should be able to take all necessary steps for the protection of the milk supply from contamination."

### THE BARNET OLD AGE PENSION CASE.

MUCH comment has been aroused in the Press and elsewhere by a recent case in which an old age pensioner was deprived of his pension because he had for a period of three months received treatment, at the expense of his relatives, in a pay ward of the Wellhouse Hospital, an infirmary belonging to the Barnet Board of Guardians. The Ministry of Health on appeal upheld the decision of the local Pensions Committee, and their action in doing so has been the subject of adverse criticism—as it appears to us, with singularly little justification. We do not know whether in the opinion of the Ministry an old age pensioner who is a paying patient in an infirmary ought to continue to receive his pension or not; but when Parliament has entrusted a Government Department with the duty of deciding appeals under a statute as a judicial tribunal, we are quite certain that it would be in the highest degree improper for the Department to give decisions based upon what they think the law ought to be and not upon what it is. By Section 3 (1) of the Old Age Pension Act, 1919 (which replaced an analogous Section in the Act of 1908), a person is disqualified from receiving or continuing to receive an old age pension if he is an inmate of any poor law institution, though in the case of a person receiving medical or surgical treatment the disqualification does not become operative for three months. The Section does not say that receipt of poor law relief is to disqualify; it disqualifies every "inmate of a poor law institution"; and these words appear on their ordinary grammatical construction to cover all inmates, whether in paying wards or not. Hence we doubt if the Ministry, in their judicial capacity, could possibly have decided otherwise. The criticisms which have been passed upon them have ignored the fundamental distinction between decisions given in an administrative, and those given in a judicial, capacity. The practice of entrusting administrative bodies with judicial functions at all may properly be objected to; but that is no reason why they should be attacked for refusing to allow themselves to be influenced by administrative considerations when acting as a judicial tribunal. We might add also that we find some difficulty in understanding how, apart altogether from Section 3 (1) of the Act of 1919, a person able to provide a sum of two guineas a week for his maintenance in a hospital is in a position to allege absence of means sufficient to justify a claim for an old age pension. We gather, however, that this was not the point on which the decision in the Barnet case actually turned.

But while the Ministry of Health, so far as we can judge, have decided strictly in accordance with the law, we do not mean to imply that the law itself may not need amendment. We think it does, but this is a matter for Parliament and not for the Department. The legislature presumably created the disqualification of the Act of 1919 because it was thought undesirable that a person should continue to receive a pension from the public purse at a time when he was an inmate of a Poor Law institution and therefore (as would normally be the case) being supported by the local rates. At the time when the Act of 1919 was passed pay wards in Poor Law infirmaries were practically unknown, and it is unlikely that Parliament ever had in mind the possibility of a case such as the Barnet case. Where, however, a person receives medical treatment in an infirmary on a commercial basis and without any charge to the rates there is no reason at all why he should be the subject of any disqualification, and the law should be amended accordingly. Nevertheless the subject is full of difficulties. The legal right of a Board of Guardians to establish pay wards at all is, we believe, by no means beyond doubt; and the whole position requires to be reviewed and, if necessary, regularised. We should indeed prefer that if pay wards are to be provided out of public moneys at all they should be provided by the public health authorities (who already possess the necessary legal powers under the Public Health Acts), and not by those of the Poor Law; but, from a practical point of view, it is obviously better to make use of existing machinery, especially in view of the large number of empty beds at present to be found in Poor Law infirmaries.

---

### THE NATION IN STATISTICS.

THE annual report of Mr. S. P. Vivian, Registrar-General for England and Wales, for the year 1920, reveals that that was indeed the wonderful year.

More babies were born than ever before since civil registration of births was instituted. The total was 957,782. The death-rate of 12·4 per thousand was the lowest recorded, and the number of deaths, 466,130, was the lowest since 1862, when the population was little more than one-half that of 1920. The number of marriages, 379,982, was the largest recorded in one year.

There were also a number of other records. The deaths from tuberculosis were considerably fewer than those of any previous year. Infant mortality reached the lowest rate of 80 per thousand births. The number of divorces obtained was nearly double that of the previous year and nearly treble that of any earlier date. As well as providing a series of revelations of a "bumper" year, the report contains many shrewd inferences bearing upon the state of public health.

It is interesting, for example, to note that nearly all the decline recorded in the death-rate of children under one year of age occurred during the second half of the calendar year, the winter quarter especially proving fatal to babies. The increase in the standardised rate of deaths from cancer was confined to those aged 45 and upwards. There are many indications that overcrowding is directly responsible for bad health. For instance, Wigan had the highest death-rate from measles, of 1,188 per million.

Out of so extensive a mine of figures, covering 531 pages of close, tabulated statistics, it is only possible to extract a few of the main facts. Even so, the cold tables are difficult to translate into the human story of social progress that they reveal. The marriage figures, for example, explain much of the social disturbances due to the war. The number of widows under the age of 25 who married again rose to a proportion of 1,071 in 1919 and to 630 in 1920, as compared with the figure of 100 in 1911. After 1916 the rates for the marriages of young widowers and elderly bachelors actually doubled. The number of divorced men marrying spinsters increased from 300 in 1911 to 981 in 1920. The figures also confirm the old belief that the balance between males and females destroyed by a war is restored by nature. The ratio of births of boys in 1920 as compared with girls was 1,052 as against 1,000.

Medical men will turn to the figures showing the causes of death. Deaths from malaria increased from 62 in 1916 to 250 in 1920, but the majority of these were undoubtedly due to infection incurred abroad. Deaths from pneumonia were the lowest recorded for the last 15 years. There was a serious increase in the number of women dying in childbirth, almost entirely due to septic causes.

Deaths in 1914 associated in certification with alcoholism, if taken as 100, in 1920 numbered for men 45 and for women 22. During the year 1918, when there was the greatest control over the liquor traffic and the largest number of persons absent on war service, the proportion was 23 for men and 14 for women. Mr. Vivian states that "it seems impossible to avoid associating this remarkable movement with the institution of war restrictions upon the sale of liquor, followed later by their relaxation."

The price of this volume is £1. This is prohibitive for many, but it is understood that the Registrar-General intends next year to change the scope of the report for England and Wales and also the manner of its presentation. If this leads to a smaller book and the issue of the report in less costly volumes, the change will be welcomed by all who study questions of public health.

---

### LEAD POISONING FROM BEER.

WE learn from the "British Medical Journal" that a considerable number of cases of lead poisoning have occurred in certain districts in Middlesex, which were traced to the consumption of beer supplied by a particular firm of brewers. A medical practitioner recognised an outbreak of lead poisoning amongst his patients and notified the medical officer of health. The latter found that the outbreak, which was fairly widely spread in his district, was limited to persons resorting to certain public-houses, in the cellars of which large iron tanks lined with white enamel had recently been provided for storing the beer. Samples of beer were found to contain about 1 grain of lead per gallon. The beer as it left the brewery was free from lead. A lead glaze had been used in the preparation of the enamel, and from this source, owing to the solvent properties of its sugars, the beer became contaminated. The use of these tanks was at once discontinued. The beer was drunk for some two or three weeks before the characteristic symptoms were produced. It is thought probable that cases of lead poisoning ascribed to unknown causes have occurred among persons who resided in other districts and frequented public-houses in which the contaminated beer was sold. During the investigation it was found that another brewery company substituted tanks for barrels in their public-houses, but in this instance the tanks were of earthenware lined with leadless glaze, and no lead was found in samples of beer taken from them. The occurrence shows the value of prompt action on the part of practitioners in reporting to the medical officer of health cases of illness which may be due to articles of food or drink. The medical officer of health should promptly inform the Ministry of Health of such cases, as the same causes may be operating simultaneously in widely separate districts, and only by co-ordination through the central authority can expeditious action be taken.

### DOCTORS' FEES IN GERMANY.

IN Berlin the practice is being adopted of charging the foreigner fees on a sliding scale adjusted to the rate of exchange in the country from which he hails. This system appears to be applied only to "valuta-rich" countries. To be strictly logical, doctors in Berlin should apply their system to "valuta-poor" countries such as Austria, but on this point discreet silence is maintained. In the waiting-rooms of Berlin doctors with a foreign clientèle the following quadri-lingual notice is prominently exhibited: "To our Foreign Patients! In conformity with the views of foreign doctors, Berlin doctors demand of their foreign patients coming from valuta-rich countries the same fees for their services as are customary in their own countries. Foreigners resident and employed in Germany are exempt from this rule." It appears that the medical profession in Berlin has obtained information from various countries as to the scale of medical fees fixed by general usage for different classes of patients and ailments. The differences in the charges of general practitioners and specialists are also noted, and, armed with all this information, Berlin doctors are advised by their union to settle the amount of their charges before treating the patient, who is expected to pay in German currency the equivalent of the fee he would have to pay in his own country. The point which the German medical profession seems to have overlooked is that many, if not most, of the foreigners at present visiting Germany have gone there because they wanted to live cheaply and thought they could do so because Germany is "valuta-poor." So if all other classes in Germany follow the course adopted by the medical profession towards foreigners the chief inducement for visiting Germany and, incidentally, bringing money into Germany, would fail, and the goose which laid the golden eggs would take wing. The only good thing to be said of this policy is that it is carried out with perfect candour; the visitor is left in no doubt as to what he will have to pay, and if he does not like the scale of fees, and his illness is not urgent, he can leave the waiting-room by the door that he entered. Appreciating the German candour, we would inform our countrymen what to expect before a ticket to Germany is bought and a holiday planned on the assumption that a "valuta-poor" country is a cheap one to visit.

### GOVERNMENT RESEARCH.

APART from the work conducted by the Medical Research Council, the amount of research now being carried on by the Ministry of Health, either directly or in conjunction with public health officers throughout the country, is not generally realised. A valuable report was recently made by a committee inquiring into the relations between smallpox and alastrim; the conclusion was that alastrim is not to be differentiated from smallpox in a modified form. Arrangements may be made for inquiry into the Spahlinger method of treating tuberculosis as soon as a sufficient supply of serum is available to allow of a thorough test. Inquiry of a highly technical nature has been made into encephalitis lethargica, and a special report by Dr. Allan C. Parsons is now available. The report giving the results of research into pneumococcus has already been issued. It is understood that, with the co-operation of a number of insurance practitioners, a survey is being made with regard to the ravages of rheumatism. The mortality from rheumatism is greater than is generally supposed. The latest official figures show that the rate of deaths from gout and osteo-arthritis was 56 per million living, and the same proportion of deaths from chronic rheumatism. The work already accomplished under the supervision of the Ministry of Health at the pathological laboratory of the University of Bristol into the conditions and forms of food poisoning is referred to in another part of this issue, but undoubtedly the inquiry into botulism was considerably facilitated by the research arrangements instituted by the Government Department responsible in August of last year.

### THE FUTURE OF SANATORIUM INDUSTRIES.

THE days when sanatorium treatment consisted of a rest cure in the open air begin to seem remote, and the scheme of graduated labour that succeeded it has developed industrially no less than in the interests of treatment. The ideal is that sanatoria, to use the word loosely, should become as nearly self-supporting as possible, on the very interesting lines that we described last month in connection with the Cambridgeshire Tuberculosis Colony at Papworth. Though Sir Clifford Allbutt explained that this invaluable village settlement was not to rival the sanatorium, it should be remembered that patients in all stages of disease are admitted, and their families so far as the number of cottages allows. The workshops are self-supporting. A somewhat similar movement, on less ambitious lines, is, of course, permeating the sanatoria. Kelling Sanatorium, Norfolk, is a convenient example. There several different industries have been started for the patients in Class I, to enable them to add to their incomes on discharge, or even to earn their living in an occupation suited to their health. The patients are encouraged to record their successes and failures, and the sanatorium receives regular correspondence from them. The outside public is invited to give orders to the sanatorium's industries. It is clear that a useful beginning has been made, but it does not seem to us that the more or less individual efforts of the patients can hope to solve the problem in this isolated way. There can be little doubt that of the three objects at Papworth, treatment, after-care and productive industry, the two latter benefit greatly from the concentration of numbers that the colony system allows. The question is whether these advantages cannot be gained to some extent by a system of co-operation between sanatoria. Such grouping has everything to recommend it—surroundings, after-care, industry in common, productivity, markets. All these are secured by co-operation of groups, and largely, if not altogether, lost when patients separate on their discharge to scattered homes and individual occupations. It is true that St. Dunstan's has devised a system of superintendence for its blind ex-patients which gets rid of most of these difficulties. But nothing of the kind has been attempted with equal thoroughness by sanatoria. We advocate such a scheme, which could be tested if the different sanatoria combined to carry itout.

### FACTORY INSPECTION AND WELFARE WORK.

THE annual report of the Chief Inspector of Factories and Workshops states that during 1921 92,565 accidents (951 fatal) were reported, as compared with 138,773 (1,404 fatal) in the previous year. This remarkable drop is almost entirely due to phenomenal inactivity in industry throughout the year and the prolonged coal strike, which resulted in many large iron mills and blast furnaces being completely closed down for an extended period. As these industries are heavy accident-producers, their closure alone would seriously affect the total figures. The number of accidents due to electricity was 322, of which 32 were fatal—a reduction of 81, or 20 per cent. on the total, and 50 per cent. on the fatal cases, compared with each of the two preceding years.

Welfare work in factories and workshops has held its own in face of adverse circumstances. Whereas before 1914 the number of factories in which such schemes were in operation was comparatively few, now the reports from the inspectors all over the country give examples which comprise, in addition to canteens, cloak-rooms, surgeries and rest rooms —matters dealt with in Welfare Orders—a large number of social activities, savings clubs, benevolent funds, life insurances, game clubs, concerts and dance meetings, summer camps in charge of athlete trainers, bowling greens, dental clinics, libraries, works' schools (from one of which several young persons are sitting for the London Matriculation), and lectures on business management, with quotations from firms' own books, are all mentioned in reports.

During the year there were 230 cases of lead poisoning, of which 23 were fatal. In 1900 the number of cases was 1,058, of which 38 were fatal. There were no cases of phosphorus or mercury poisoning last year, but one case of arsenic. The lower figures for anthrax poisoning (25, with six fatal) are held to be partly due to the inauguration of the Government station for the disinfection of wool at Liverpool. Owing to trade depression this station is to be temporarily closed at the end of September.

On the question of hours of work, it is stated that since the 48-hour week has been continued over a considerable period output tends, in the majority of cases, to attain the old level, and that the beneficial effect of increased leisure on the workers is becoming apparent. The plan of "no work on Saturday" appears to be increasing in favour.

### THE FUMIGATION OF SHIPS.

AN important inquiry of considerable interest to the shipping world and to port sanitary authorities has just been completed by Dr. R. J. Reece. In consequence of two deaths at Southampton, due to the use of hydrocyanic acid gas in fumigating a well-known liner, a shipping company asked Sir George Newman to lend experts to inquire into the precautions to be adopted when this highly poisonous gas was used. Until the end of March, sulphur was employed for fumigating ships. The United States Public Health Department has now demanded that our largest Atlantic liners trading between the States and all foreign ports shall be fumigated in all parts at the same time every six months. This includes all shipping. The use of sulphur is specially unsuitable for passenger liners, as it bleaches, attacks metals, deteriorates furniture and decorations, and leaves behind it an unpleasant smell. Despite the danger of HCN, owing to the fact that it has not a pungent smell, it has had to be adopted. At Liverpool, Dr. E. W. Hope, the Port Sanitary Officer, used one barrel of the chemical for every 45,000 cubic feet of space. Before the final steps of generating the gas were taken, he ascertained that every man was accounted for. Nobody was allowed on board after a certain time, and when the ship was opened up it was left for a time in order that the gas might clear. Finally, the Assistant Port Medical Officer, protected by a self-contained oxygen breathing apparatus of the Salvus type, and carrying a cage of rats, went below in order to test the atmosphere. Only when the rats had been exposed for a few minutes in every part of the vessel was anyone allowed on board. This method is likely to become the routine for the future.

### LABOUR AND HOUSING.

AS a preliminary to the Trades Union Congress, the National Housing Conference arranged by the Labour Housing Association was held at Southport. Mr. R. B. Walker, President of the Trades Union Congress, who presided, criticised the Government in strong terms for their failure to redeem their pledges in regard to housing. He characterised their action as "sheer stupidity, combined with shameful neglect of the interests of the vast numbers of men." We consider the description well deserved, but for a reason which will be anything but acceptable to Mr. Walker. We should have thought that a trade-union leader would have been ashamed to mention housing, for it is to the methods inculcated by trade unionism, more than to anything else, that the housing fiasco of the Government is due. Has Mr. Walker never heard of bricklayers who did not lay more bricks than they could help? When the Government undertook housing they showed the business incapacity which has characterised them in other directions. They created an immense artificial demand for material and labour in the building trade, of which the interests concerned immediately took advantage. Material and labour soared to fabulous prices. The men seized the opportunity of doing the least possible work for a high wage, thus further increasing cost. The result was that the expense became too great even for a Government of unprecedented prodigality. The appalling waste of the scheme can be judged by one fact. According to the "Times," an organ by no means unfriendly to labour, bricklayers are now laying twice as many bricks as they did, and yet they are by no means exerting themselves unduly. This policy of unenlightened selfishness has been pursued by organised labour in every direction, with consequences that any economist could have foretold. But we are a "practical people," who appear incapable of learning in any but the dear school of experience, which the proverb says is kept for fools.

### ANTITOXIN AND DIPHTHERIA.

THE persistent high incidence and high mortality of diphtheria has caused the Ministry of Health to draw attention to questions relating to the supply and use of diphtheria antitoxin. The Diphtheria Antitoxin Orders issued by the Local Government Board in August, 1910, authorised local authorities to provide medical practitioners with antitoxic serum for the prevention and treatment of diphtheria, and required that the arrangements with respect to the keeping, distribution, and use of the diphtheria antitoxin should be made in accordance with the advice of the Medical Officer of Health.

The circular letter which accompanied these Orders referred to the importance of promptness in antitoxin treatment. The arrangements should be such as to secure that the medical practitioner who needs antitoxin from the local authority can always obtain it without delay. Moreover, the local authority can help to educate the general public regarding the insidious onset and the danger of diphtheria, and so induce them to obtain early diagnosis and treatment.

The present Memorandum adds that early administration of antitoxin not only reduces the mortality from diphtheria, but causes the disease to run a milder and shorter course; while by lessening the period of the patient's infectivity to others it reduces the period of occupation of a bed in an isolation hospital. It is thus an important measure in the interests of economy as well as of the public health. It should be the aim of every medical practitioner, so soon as on clinical evidence he has diagnosed diphtheria, either to have the patient removed forthwith to an infectious diseases hospital or himself to administer an adequate dose of antitoxin.

### A COLOUR SCHEME FOR POISONS.

RECENT cases where patients have died from being supplied with poison, instead of less harmful medicine, led Mr. Justice Darling to make the suggestion that all liquid poisons should be of a definite colour, so that they would be unmistakable for non-poisonous preparations. This suggestion, which appears at first sight to be practicable, has been considered by the President of the Pharmaceutical Society, who takes the view that, while special precautions may be necessary to ensure safe dispensing, the human factor must always be reckoned with. There is a Pharmacy Act Regulation which enacts that all poisons shall be kept in bottles distinguishable by touch. If this was rigorously observed in shops, dispensaries, and especially in doctors' surgeries, some of the mistakes might have been avoided. Mr. Justice Darling's suggestion in practice might easily lead to other serious mistakes. For if all poisons were coloured blue, for example, there might easily be confusion between the deadly poisonous corrosive sublimate, and morphia solution. The only real precaution would appear to be that there should be no lowering of the standard of qualification for dispensers. The Codex Committee of the Pharmaceutical Society recently reported that in their opinion, where a substance can be coloured without detriment to its properties, this should be done. But to be effective colours must remain of a permanent shade, and the colouring matter used must be inert, otherwise analysis would be rendered difficult. It is understood that experimental tests are now being carried out, in the first place with solutions of strychnine B.I., in order to ascertain how solutions can be coloured effectively without harming the substance.

### HOSPITALS AND SUNDAY GAMES.

THE recent decision of the London County Council to allow games to be played on Sunday afternoons in the parks and public places under its control gives a new interest to the periodic suggestion that hospital sports and entertainments for their benefit should be allowed on that day of the week. It appears that when it was announced that the Leeds Hospital Sports were to be abandoned, the proposal was made that they should be held on a Saturday and Sunday. But the plan had critics in Leeds, despite the assertion that the patronage of such charitable events is a charitable act. Probably the North of England is more Puritan in its views on these matters than are the home counties; and if the example set by London is followed, as we may expect, elsewhere, there seems no reason why hospitals should not organise Sunday fêtes with less serious people. There is also this to be said; that organisers of charitable fêtes, which are often planned at short notice, sometimes find that no suitable weekday is available; and now that entertainments of the kind are a recognised and regular source of income, the work of a deserving charity may suffer if a fête on its behalf cannot be held. In these matters no hospital desires to overstep or lag behind the scruples of the decent people who support it, but now that Sabbatarianism is waning and secular amusements are allowed between church hours, is there any reason why hospital sports should be excommunicated? We think there may soon be a general assent.

